# Main Therapeutic Uses of Some Moroccan Hot Springs' Waters

**DOI:** 10.1155/2021/5599269

**Published:** 2021-06-21

**Authors:** Kawtar Fikri-Benbrahim, Asmae Houti, Abdelhakim El Ouali Lalami, Rachid Flouchi, Naoufal El Hachlafi, Mariam Houti, Saad Rachiq

**Affiliations:** ^1^Laboratory of Microbial Biotechnology and Bioactive Molecules, Sciences and Technologies Faculty, Sidi Mohamed Ben Abdellah University, P.O. Box 2202, Fez, Morocco; ^2^High Institute of Nursing Professions and Health Techniques of Fez, Regional Health Direction, El Ghassani Hospital, Fez 30000, Morocco; ^3^Engineering Systems Laboratory, National School of Applied Sciences (ENSA), Ibn Tofail University, Kenitra, Morocco; ^4^Laboratory of Functional Ecology and Environmental Engineering, Sciences and Technologies Faculty, Sidi Mohamed Ben Abdellah University, P.O. Box 2202, Fez, Morocco

## Abstract

Morocco has an important groundwater reserve, especially in the Atlas domain, corresponding to its largest water reservoir. This reserve comes from rainwater infiltrated into rocks and sediments to give rise to mineralized waters feeding many springs and having curative properties, which confer each spa-specific therapeutic indications, based on the medicinal properties of its waters. All over the world, mineral waters of thermal springs have interesting therapeutic uses to cure some diseases; unfortunately, such potential is underexploited in Morocco. This narrative review deals with the distribution of thermal springs in Morocco, the classification of their thermal waters, and their health effects, with the aim to enhance them. For this purpose, previous studies' results on different aspects of thermal waters were searched in the most famous scientific databases, by using targeted specific keywords. Literature has shown that Morocco contains several thermal springs, scattered throughout the Middle Atlas, in the South, and in the Rif, which are regarded as waters of high therapeutic quality, whose thermal cures are often recommended to people suffering from rheumatism and locomotor system's diseases, skin diseases such as eczema and psoriasis, cardiovascular diseases, overweight, or respiratory troubles. However, apart from the unique and real thermal station of Moulay Yacoub, the other thermal springs are exploited in an empirical and artisanal way, mainly by a local population. So, as hydrotherapy constitutes a good choice of natural therapy using water components as a complement or alternative to conventional treatments, a better understanding of the thermal springs' distribution in Morocco, their thermal waters' classification, and their potential health effects may enable their valorization through a better use of their waters.

## 1. Introduction

Water is at the life origin: in addition to its vital role as a nutrient, it is involved in many essential physiological functions such as digestion, absorption, thermoregulation, and waste elimination [1].

The level of water cycle in the terrestrial globe is approximately as follows:

- 60% rainwater which remains in the atmosphere and maintains the evapotranspiration cycle.
- 15% which flows and joins the streams.
- 25% that seeps into the ground and supplies underground aquifers. A small part of these waters will percolate to a considerable depth (−2000 m) to form mineral waters, some of which will supply spas [2].

Thermal water is a hot mineral water, endowed with therapeutic properties used in a thermal establishment thanks to a set of properties favorable to health. It contains mineral salts, gases, and sludge, which can act effectively on health. Water is considered as mineral when it comes from an identified spring. According to French regulations, this water must have the same composition and the same degree of purity at the origin and the point of use [3].

Natural mineral water and spring water are subject to strict European regulations, defining their classification as well as physicochemical and microbiological quality. Compliance with international standards requires the removal of compounds such as iron, manganese, sulfur, and arsenic by decantation and/or filtration, possibly accelerated by prior aeration. The application of this separation should neither modify the water composition in its essential constituents nor aim at modifying the water's microbiological characteristics [4].

The soil's geological nature influences the filtration of meteorological water. Indeed, during its infiltration, it is charged with ions and acquires physicochemical properties characterizing the corresponding water aquifer. According to El Wartiti et al. [5], groundwater is more or less mineralized depending on the following:

- The nature of rocks crossed and the minerals encountered during water infiltration.
- The contact time of water with minerals and therefore percolation rate of water in subsoil.
- The renewal time of the aquifer water by the infiltration water.

At depth, water can still be enriched with CO_2_ or H_2_S gas [6]. Thus, the spurting of these mineral waters will be accompanied by thermal gases emission.

Thanks to their affluence in mineral components and gases, thermal waters can have therapeutic uses. Hydrotherapy constitutes a good choice of natural therapy using water components as a complement or alternative to conventional treatments.

In Morocco, thermal springs are largely widespread thanks to the importance of its groundwater reserves; however, the therapeutic potential of thermal waters is underexploited.

Although numerous studies reported some therapeutic properties and uses of some mineral waters in Morocco, to the best of our knowledge, no review was published to summarize these reports and suggest scientific-based therapeutic applications of these waters. Therefore, this review was designed to critically summarize all published works on physicochemical composition of a natural mineral water to give them scientific therapeutic properties and allows distinguishing recommendations and limits of their use clearly. Moreover, this review offers a better understanding of spas distribution and of their thermal water's classification and health effects aiming at valorizing them through a better use of their waters based on scientific evidence.

Hence, the current paper aims to provide data support and prospect concerning future applications of hot springs' waters in Morocco as efficient complementary therapy.

## 2. Geographical Distribution of Hot Springs in Morocco

Morocco has a nonnegligible groundwater that feeds various thermal springs, either at the level of Jurassic carbonate formations of the southern Rif wrinkles (SRW) or in the marly-dominated tertiary formations of the Rif domain and Southern Rif furrow. These hot springs constitute a hydrothermal system in the western part of a belt oriented East-West going from Morocco to Tunisia [7].

Thermal waters' natural emergence in Rif region, linked to networks of conjugate faults, implies the existence of open fracturing within surrounding rock and permanence of this opening. This fracturing, linked to tectonic evolution of northern Morocco, results from combined effects of the convergence of Africa towards Eurasia and the movement towards southwest of Alboran plate.

Northeastern Morocco is distinguished from the Rif by a succession of horsts and grabens. While, in grabens, the Liassic limestones, constituting an aquifer, can be buried to depths of the order of five km, in horsts, these limestones, often fractured, occur at altitudes of about 1200 m, allowing the meteoric water infiltration [8].

**Figure 1:**
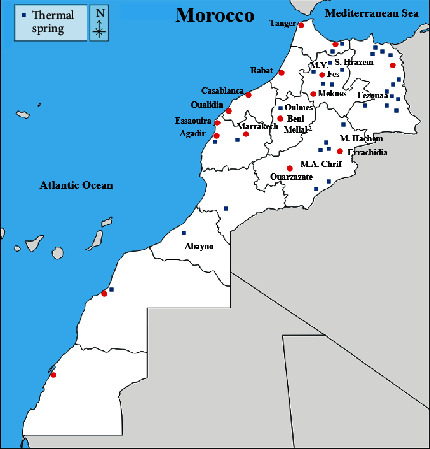
Geographical distribution of the most famous hot springs in Morocco.

Morocco has almost 120 thermal springs spread over six thermal spas areas: Northeast, Rif and South-Rif, Center, Middle Atlas, High Atlas, and Anti-Atlas and Sahara (Figure 1). More than 50% of these sources belong to the Rif, pre-Rif, and South-Rif zones [9], and more than twenty are very famous.

These thermal waters have a first quality therapeutic potential; indeed, some waters of the South Rif corridor present a set of physicochemical characteristics and contain trace elements and macro elements summarized in Table 1, which give them beneficial health properties [10]. The Thermal gases produced during the gushing of some thermal waters can in turn have their own therapeutic properties on certain diseases, in particular respiratory and cardiovascular diseases, and phlebology.

### 2.1. Saïss or the Pre-Rif

The Saïss basin is a large asymmetrical Syncline, having an East-West direction, and is part of the South Rif furrow. It is bordered by pre-Rif wrinkles to the north and outcrops of the middle Atlas to the south. In the west, it is bounded by Oued Beht valley and its Paleozoic outcrops which are part of the Western Meseta, and finally to the east by Touabar pass which separates the basin from the Fez-Taza corridor [11]. This basin contains some of the most famous thermal springs in Morocco due to their beneficial health effects.

  Sidi Harazem Station: this thermal station, discovered in Roman times, is located about 30 km east of Fez city (34° 1′33 63″N 4° 52′ 55″ O, 728 m of altitude). It is a source of calcium-rich magnesium bicarbonate water, which meets Moroccan drinking water standards [12, 13], and that has been the first mineral water bottled and commercialized since 1968. This station is renowned for curative properties of its drinking water in the treatment of liver, intestinal, and digestive diseases as well as renal lithiasis [14].
  Ain Allah: it is a thermal spring located 15 km northwest of Fez city and consists of a drinking water fountain, jet showers, and two large swimming pools open for public swimming. This station's source is extracted by artesian drilling at 1650 m depth located in Douyet domain (34° 3′ 0″ N, 5° 6′ 36″ W).
  This water meets Moroccan drinking water standards and contains fairly high levels of some major elements (Ca^2+^, Mg^2+^, Na^+^, and K^+^). Thereby, the composition of calcium in Ain Allah's water can offer it therapeutic interests in the treatment of some dermatological pathologies and also in the intracellular calcium regulation in keratinocytes [13].
  Moulay Yaâcoub: it is located 25 km northwest of Fez city, at 34° 5′ 28.00″ N and 5° 10′ 58′ 50″ W, and it is the most famous Moroccan thermal station which receives more than 40,000 people per year. Hence, it generates a turnover of 6,400,000 Dhs: 4,000,000 Dhs for the traditional baths and 2,400,000 Dhs for the new establishment in 2007 [15].

The Moulay Yaâcoub hydrothermal complex covers an area of 300 ha and includes jet showers, a women's swimming pool, and two other large mixed swimming pools. It comprises a main griffin and six secondary resurgences, with a flow rate exceeding 70 L/s at the source. The volume of rocks full of water exceeds 500,000 m^3^. Its thermal waters circulating at depths between 1.2 km and 6.4 km, within the Miocene marls of the South Rifain strip, are smelly and have double chemical facies: chloride sodium, chloride calcium, and magnesium. They show high contents of metal trace elements similar to those of hot springs' water flowing through crystallophyllian formations. These waters may also contain other elements such as sulfate, sodium, bicarbonate, and calcium [7]. Hence, such waters are recommended for rheumatic diseases and osteoarthritis treatments as well as for skin diseases (acne, eczema, psoriasis, etc.) treatment [16] and are well known for their effects on respiratory tract diseases.

Due to the H_2_S toxicity, only few bacteria and microorganisms can survive in this water. Thereby, bactericidal and antifungal activities of these thermal waters have been reported with particular emphasis on dermatological diseases. Thus, this specific bactericidal activity can be of particular importance in the management of thermal waters [17].

Great importance has been devoted to pH values and the presence of specific anions or cations in water. H_2_S may be present under certain conditions as different reactive sulfur species. For example, the interaction between sulfur and oxygen radicals leads to the formation of byproducts such as pentathionic acid (H_2_S_5_O_6_), which may be at the origin of antibacterial and antifungal activities of thermal water on skin.

The thermal springs of Moulay Yaâcoub are considered hyperthermal waters because they spring at temperatures between 51°C and 58°C. Hence, the water is cooled down to 38°C before being used in the thermal centre. These nondrinkable waters, at neutral pH (6.8 < pH < 6.6), are also characterized by high electrical conductivity (*C* = 95.7 ms/cm). Their gaseous composition is dominated by nitrogen (46%), followed by methane (42.5%), carbon dioxide (9.9%), and ethane (1%). Oxygen, argon, and hydrogen are present as traces [7].

The different cure techniques mostly used for these waters are detailed at the end of this review.

  AinTratt: this source is located at the foot of Tratt mountain southern slope (Tratt: 34° 3′ 25.014″ N, 5° 1′ 54.895″ W, 406 m), 3 km NW of Fez city. On the map of Fez-west at 1/50,000, its coordinates are *X* = 533.40, *Y* = 386.25, and *Z* = 540 m. This source is characterized by a rather low water temperature and an average mineralization characterized by chloride sodium facies and carbonate subfacies [18].
  Zalagh: this thermal spring is located at the foot of Zalagh mountain northern flank (34° 6′ 23″ N, 4° 58′ 4″O, 835 m), about ten kilometers NE of Fez city. Its coordinates on the topographic Fes-east map at 1/50,000 are *X* = 545.15, *Y* = 392, and *Z* = 510 m. Zalagh thermal water has a sodium chloride facies with a sulfate calcium tendency, showing the influence of the evaporate Trias on this water's circulation [18]. Hence, this thermal spring's water is characterized by a chlorinated sodium chemical composition with an H_2_S release and a high temperature.
  Ain Salama: discovered in 1985, this source located in an attractive geographical region, 13 km from Meknes, is a part of Meknes uplands occupying the western part of Meknes-Fez basin (33° 52′ 22.85″ N, 5° 32′ 26.62″ W, 497 m), overlooking Oued El Kellet, and presents several therapeutic, curative, and touristic benefits. It includes an uncovered swimming pool and two baths with individual showers. The mineralized water gushing at 39°C and characterized by a high electrical conductivity (close to 2000 *μ*S/cm) is, therefore, highly mineralized and recommended for drinking to reduce stress, increase energy, revitalize blood circulation, relax muscles, and reduce toxins.

However, it should be consumed with moderation, especially for people suffering from severe heart failure or labile hypertension, while it is strictly forbidden to infants whose organism cannot adapt to mineral overload [11].

### 2.2. South Rif Ripples

The southern Rif ripples form the most southern part of Rif range and correspond to a depression extending from Atlantic in the west to the Taza Strait in the east.

  Ain Hamra: also named Ar-Rahma, it is located 1 km from the provincial road connecting Aknoul to Boured and not far from Ajdir center in Taza province (3° 30′ 0″ N and 7° 0′ 36″ W). Rich in iron, its water, similar in its taste and flavor to the famous French Vichy thermal water; is known for its curative properties on anemia, respiratory diseases, liver and digestive diseases, rheumatic diseases, and joints aches [19]. Thanks to these benefits, this thermal spring records more than 4000 spa guests, especially during the summer and spring periods, according to the rural municipality of Ajdir [19].
  Outita: located 12 km from Sidi Slimane town, on the road to Khemissat, its Lambert coordinates on El Kansera map (1/50,000) are *X* = 459.60, *Y* = 392.7, and *Z* = 120° (34° 9′ 46″ N, 5° 45′ 46 ″ W).
  The source is characterized by an average temperature of 40°C and a chloride chemical facies with very high mineralization [18]. Waters having the characteristics quoted above have an effect in rheumatology and dermatology fields.
  Ain Boudra: located at the bottom of Jbel Boudra western slope, at 2 km SE of Sidi Kacem town, this source has the following Lambert coordinates: *X* = 472.6, *Y* = 400.6, and *Z* = 185 m on Sidi Kacem topographical map (1/50,000). It is characterized by calcium bicarbonate chemical facies with low mineralization and a temperature not exceeding 25°C [18].

Bicarbonate waters are the most diffuse in nature thanks to the widespread concentration of bicarbonate over calcium, sulfate, sodium, and magnesium in the soil. These waters come by infiltrating into a calcium soil. Calcium and magnesium bicarbonate are known from the reaction with CO_2_ that is generally present in both volcanic soils (deep origin) and atmosphere. Bicarbonate waters are used in therapy to cure cardiovascular and respiratory diseases [14].

  Tiouka: this source is located 6 km north of Ain Boudra; it emerges in the Tortonien sandy marls, not far from its contact with the southern Rif complex (Bab Tiouka: 34° 14′ 36.775″ N 5° 39′ 46.389 W, 72 m). Its coordinates on Sidi Kacem map (1/50,000) are *X* = 475.4, *Y* = 404.3, and *Z* = 75 m. This source is characterized by a low temperature (24°C) and a chlorinated sodium chemical composition [18].
  Moulay Driss: this source is located in Oued Kroumane valley, which crosses Moulay Idriss Zerhoun town (*X* = 490.25, *Y* = 383.5, and *Z* = 540 m on Sidi Kacem map (1/50,000)). Its geodesic coordinates are 34° 3′ 21,247′ N, 5° 31′ 15,783″ and 550 m. From a stratigraphic point of view, Moulay Idriss thermal spring emerges in the middle and upper Lias series belonging to Fert El Bir ride. It is fed by infiltrating waters at an altitude of about 948 m (calculated by isotopic method) [18].

With a temperature exceeding 32°C, the Moulay Idriss source is characterized by a chemical composition rich in sulfate [18]. In sulfated waters, the main element present is the sulfate ion (SO_4_^2−^). Other elements such as bicarbonate, calcium, magnesium, chloride, and arsenic could also be found.

These waters are particularly suitable to treat liver, kidney, gastroenteric, and respiratory diseases [14].

### 2.3. Eastern Morocco (Oriental)

Eastern Morocco, designated in this work, is bounded on the west by Oued Mouloya, on the north by the Mediterranean Sea, and on the south by high uplands and on the east by Algerian-Moroccan border.

 
*Fezouane*. This thermal resort is located 2 kilometers halfway from the main road connecting Ahfir and Berkane, in the Fezouane rural commune located at the foot of Beni Znassen's mountains on an area of 210 hectares (34° 54′ 51″ N, 2° 11′ 56.67″ W, 272 m above sea level). Discovered in 1950 approximately, it receives more than 10,000 visitors each oriental region and other Moroccan regions, each year, thanks to the quality of its warm and soft mineral waters recognized by their therapeutic effects [15]. Indeed, a preliminary study has demonstrated the therapeutic effect of its thermal mineral waters for some renal lithiasis [20].

### 2.4. High Atlas and Errachidia Region

  Hammat My Ali Cherif: this spa resort is located 40 km from Errachidia towards Meknes and 20 km from Er-Rich city (32° 11′ 0.866″ N, 4° 21′ 54.664″ W, 1226 m Altitude) and is equipped with pools and sanitary blocks. According to one Ministry of Public Health's study on the therapeutic use of this spa resort water, it is recommended in the following cases: constipation and intestinal atonies, body detoxification, obesity, and some rheumatic pains and arteritis [2].
  Moulay Hachem Thermal Spring: it is located 4 km from Hammat Moulay Ali Cherif and 15 km from Er-Rich city in Tafilalet region (32° 15′ 35.436″ N, 4° 23′ 36.158″ W, 1279 m of altitude). Its water is recommended for digestion [2]. Moreover, this cold spring water (27°C) is used to treat kidney, urinary tract, and skin diseases as well as digestive disorders.

Both of these springs are frequented by national and foreign visitors to warm up, heal a cold, and reduce winter weariness; thanks are due to their water's quality and therapeutic virtue are acknowledged.

### 2.5. South

  Abaynou: it is located on the southern flank of the western Anti-Atlas, 200 km from Agadir, and 15 km NE from Guelmim (29° 5′ 51.4″ N, 10° 0′ 59.11″W, 424 m altitude). It represents a tourist destination complementary to the other region's sites such as Ksours, oasis, and beaches.

The physicochemical analyses carried out in this station water have shown its interesting therapeutic characteristics for dermal and rheumatic diseases; its richness in components such as calcium, chloride, magnesium, sodium, potassium, sulfate, and CO_2_ is acknowledged [21].

## 3. Classification of Moroccan Thermal Waters

The classification of thermal waters can be done according to different criteria such as water mineralization and its physicochemical composition [22].

Mineralization is constant over time and represents the total amount of dissolved salts. Mineral waters can be classified according to Roques [22] as follows:

- Very low mineralized waters: with mineralization rate (MR) less than 50 mg/L
- Low mineralized waters: mineralization rate between 50 and 500 mg/L
- Medium mineralized waters: 500 < MR < 1000 mg/L
- Mineralized water: 1000 < MR < 1500 mg/L
- Highly mineralized waters: MR > 1500 mg/L

On the basis of ion composition, mineral waters can be classified as follows:

Bicarbonate waters, sulfated waters, chlorinated waters, sulfurous waters, and so forth.

The physicochemical composition of thermal waters varies greatly from one source to another, depending on travel time in the rock and the type of rock traveled; indeed, the thermal water flow to reach surface is very important, since it can modify its composition.

If water is very hot and reaches its boiling point before reaching the surface, only steam will emerge. These gases oxidize when they mix with cold waters and produce acid sources, which have a muddy appearance caused by water acidity, which corrodes surrounding rock.

The classification of thermal waters is not directly related to their calcium content; it mainly takes into account the main anion associated with them [3].

According to hydrogeochemical classification based on Piper's triangular diagram and results given in Table 1, four facies types with four different circulations can be distinguished [23].

(a) Sodium chloride facies with three subfacies:
    (i) A super chloride sodium subfamily (Cl^−^-Na^+^), represented by the following springs: Moulay Yaâcoub, Zalagh, and Tiouta
    (ii) A carbonate underframe that represents Tratt spring
    (iii) A sulfated subfacies representing Outita spring
(b) Sulfated calcium facies (SO_4_^−^ Ca^++^): characterized by strong mineralization, represented by a single spring (Moulay Driss). The circulation in this case occurs through Liasic lands with a way through evaporite rocks represented mainly by gypsum, favoring high sulfates content, unlike the other waters.
(c) Calcium bicarbonate (Na^+^HCO_3_^−^) facies, with low mineralization (Ain Allah, Ain Boudra) and circulation taking place in Liasic lands.
(d) Calcium bicarbonate facies with chloride-sodic tendency and medium mineralization represented by Sidi Harazem, Fezouane, Ain Salama, and Ain Al Hamra Spas. The circulation of these waters also takes place in the Liasic lands but with an influence of Triassic evaporite lands.

## 4. Hydrotherapy in Morocco

Hydrotherapy concerns all activities related to exploitation and therapeutic use of thermal waters. Hence, it represents the science of using mineral water sources for therapeutic, wellness, or fitness purposes. These so-called “natural mineral” waters could have a beneficial effect in the treatment of much chronic affections during spa treatments [24].

The Babylonians had already established a therapeutic system based on baths practice and applications of hot and cold water as well as ablution in rivers. Greeks and then Romans assured the later diffusion of hydrotherapy. In France, many spa towns were developed on Gallo-Roman sites. European thermalism developed much in the seventeenth century, and the nineteenth century represented the maximum development period. It was not until the twentieth century that physiological effects of immersion were described [25].

The present situation appears to be much contrasted: English-speaking countries seem to have almost completely forsaken this therapeutic approach, whereas it is still proposed in some continental European countries.

In Morocco, the thermal springs' capital is noteworthy but unfortunately underexploited, apart from a few sources used industrially for drinking (Sidi Harazem, Sidi Ali) and only one real thermal resort (Moulay Yaâcoub). So, this immense hydromineral richness is not fruitful, whereas it could be of obvious economic interest and valuable medical input [26].

Thermalism is recommended for chronic diseases treatment, especially when medical treatment becomes either insufficient to relieve the patient or too heavy to bear. Depending on the case, hydrotherapy can be considered as follows:

- Symptomatic treatment of immediate and/or delayed action
- Complementary therapy
- Therapy to withdraw medication
- Sometimes first-line therapy
- Other times a last resort when all failed

The physiological effects of baths are now well known. They are characterized by increased diuresis, cardiac output, and haemodilution, in addition to the consequent improvement in tissue per fusion and reduction of the lower limbs' edematous component [27]. Some clinical trial studies have demonstrated these therapeutic effects and showed that they are mainly related to the skin absorption of mineral elements or to skin temperature regulation [28, 29].

## 5. Health Benefits of Spa Treatments

Thermalism is generally organized around different orientations such as rheumatology, dermatology, otolaryngology or ENT, phlebology, and so forth. These orientations are mainly related to the nature of thermal mineral products as shown in Table 2 [22]. Indeed, thermal waters can have various curative effects; namely,

(i) Sulfured waters have a curative action on the mucous membranes, which allows treating respiratory tract diseases (rhinitis, bronchitis, asthma, etc.).
(ii) Sulfated waters (576 mg/L sulfates) are indicated in kidney disease. Calcium sulfate waters act on certain metabolic diseases. Calcium and magnesium sulfate waters are used to treat eczema and also to remedy the sequel and scars of burns.
(iii) Chlorinated waters (mainly sodium chloride) are indicated for the treatment of developmental disorders and enuresis.
(iv) Bicarbonate waters:
    (i) Sodium bicarbonate waters facilitate the treatment of some gastrointestinal and hepatobiliary disorders. They regulate motility of the digestive tract, attenuate digestive spasms, and have a cicatrizing action on the intestinal mucosa.
    (ii) Calcium bicarbonate waters have an anti-inflammatory, soothing, and healing effect and are used in dermatology to treat acne and burns.
    (iii) Chlorobicarbonate waters, whose ratio HCO_3_^−^/Cl^−^ = 1, are used in rheumatology.
    (iv) Oligomineral waters that are weakly mineralized (with a resistivity greater than 1500 Ω/cm at 18°C) are used in the treatment of urinary diseases [30].

Moreover, physical parameters of thermal waters, including temperature, pH, mineralization, and electrical conductivity greatly influence solubility and speed of chemical reactions affecting subsequently their potential therapeutic effects [31, 32]. Indeed, hypothermal waters are involved in many physiologic reactions, including a decrease in local metabolic activities, muscle spasm, local edema, and nerve conduction, as well as an increase in local anesthetic effects [33]. While thermal waters with highest temperature (hyperthermal waters) could be used as short-term thermal stress, human skin may liberate significant amounts of opioid peptides, leading to the modulation of the threshold of pain [34].

Furthermore, buoyancy, another physical property of water, releases a submerged body from the gravitational pull, reducing joint load [31], facilitating safer and more efficient movement, and resulting in maximum levels of exercise. Indeed, buoyancy is a force that can assist, resist, and support motion in the water [31]. Meanwhile, hydrostatic pressure, the exerted force on an immersed object, which is directly proportional to the immersion depth, could provide enhanced tactile input [31]. Viscosity, cohesion, adhesion, and surface tension can provide a graded progression of resistive exercises [31].

### 5.1. Rheumatology and Sequelae of Joint Trauma

The thermal cure contributes to a lasting improvement of pain and the function of life quality, as well as a decrease of drug consumption in general, especially the anti-inflammatory ones [35]. These indications concern mainly the following: psoriatic arthritis, osteoarthritis, fractures sequelae and bone surgery, chronic low back pain, and finally chronic inflammatory rheumatism which have been subject to studies showing in particular a significant improvement in movement and in gripping force (apart from the acute thrusts).

Indeed, the heat of the used water combined with a high concentration of minerals allows for, on one hand, an increase of the blood flow, which could have an “anti-inflammatory” action in the inflamed osteoarticular tissues and, on the other hand, muscle relaxation, thus promoting osteoarticular mobilization and decreasing the pain level [30].

The majority of Moroccan spas have rheumatology indication, the best known being Moulay Yaâcoub, Moulay Driss, Zalagh, Ain Hamra, Abaynou, Moulay Ali Cherif, Fezouane, and Ain Allah.

### 5.2. Respiratory Tract Diseases

Sulfuric waters and sodium bicarbonate waters are used for various chronic ear, nose thorax (ENT), or bronchial diseases that affect adults and mostly children, such as sinusitis, rhinitis (acute repetitive or nonallergic infections), child chronic rhinosinusitis, and nasal-sinus polyps.

Hence, these waters are indicated, for adults, to treat chronic pharyngitis, cryptic tonsillitis, or repeated angina (in case of contraindication to tonsillectomy) and, for children, to treat repetitive rhinopharyngitis, despite an adenoidectomy.

In Moulay Yaâcoub thermal spa, the best known in Morocco for respiratory diseases treatment: ENT techniques are called “specialized” because they bring “the thermal medicine” in contact with the sick zone, as opposed to other techniques known as “General” such as external balneotherapy and drinkable internal cures [2].

The main spa resorts have the otorhinolaryngology (ORL) indication, the best known being Moulay Yaâcoub, Ain Hamra, Zalagh, and Ain Boudra.

### 5.3. Cardiovascular Diseases

The mineral waters used for cardiovascular disease treatment have sodium chlorobicarbonate facies rich in free carbon dioxide.

Most cures offer care to dilate blood vessels using thermal CO_2_, a powerful vasodilator. It is called thermal therapy, carried out either by the action of heat or by the action of thermal gas that can be used isolated (dry gas) or associated with the mineral water (carbo-gas baths).

The cardioarterial diseases indication is focused mainly on peripheral arterial diseases (especially obliterative arteriopathy of lower limbs) and Raynaud phenomena and less frequently on cerebral and coronary arterial pathologies [36].

The spas with the arterial cardiac diseases indication are Abaynou and Ain Salama.

### 5.4. Phlebology

These are diseases associated with dysfunction of back circulation in the lower limbs, leg ulcers, and other trophic disorders of postthrombotic syndrome, and decompensated varicose veins constitute the most severe form [37].

Indeed, thermal cure has a significant effect on the incidence of leg ulcers and other symptoms as well as on the life quality of patients with chronic venous insufficiency [38].

The main spa resorts have the phlebology indication, the best known being Ain hamra, Abaynou, Moulay Driss, and Moulay Ali Cherif.

de Moraes Silva et al. [39] suggested that balneotherapy could improve moderately some clinical manifestations, such as pain, quality of life, and skin changes, of the chronic venous insufficiency (CVI), which affects superficial and deep venous systems of the lower limbs. However, it has no clear effect on disease severity signs and symptom score and on adverse effects on leg ulcers and edema compared to untreated cases. Moreover, Mancini et al. [40] reported that balneokinetic treatment enhances clinical and quality of life in patients with symptomatic varicose submitted to elastic compression, thanks to an associated amelioration in the venoarteriolar reflex. More recently, a randomized clinical trial study carried out by Menegatti et al. [41] reported that thermal aquatic immersion significantly improves the clinical benefits of a standardized exercise protocol for patients developing chronic venous diseases (CVD).

### 5.5. Digestive System and Metabolic Diseases

Besides beverage cure which is an essential element of internal cure, other thermal treatments can be classified in tonic care, sedatives, improving motor skills, and local healing using external cures: baths, showers, and individual care physiotherapy.

Current indications concern some so-called functional diseases:

- Digestive system diseases: intestinal functional disorders, sequelae of intestinal parasitic diseases, and chronic intestinal inflammatory diseases.
- Metabolic diseases: overweight and obesity and lipid metabolism disorders.

A previous study showed that a three-week spa treatment proved to be more effective than medical treatment for sustained and significant weight loss in 257 obese (BMI (body mass index) >30) or overweight (27 < BMI < 30) patients [42].

The main hydrothermal resorts, with this indication, have either calcium and magnesium sulfate facies (Moulay Driss, Moulay Ali Cherif, and Moulay Hachem) or calcium bicarbonate facies (Ain Boudra, Ain Hamra).

### 5.6. Urinary System and Metabolic Disorders

In this context, indications are essentially urological (rebellious lithiasis). In addition to dietary education, cures are based on beverages treatments: 2 to 4 liters/day distributed over the day and before going to bed. In fact, the mineral waters used vary according to their chemical compositions, their pharmacological properties, the pathology to be treated, and the age of the patient and his visceral state and according to the existence of any associated disease.

In the case of calcium lithiasis, calcium-rich mineral waters should be avoided. In the case of uric and cystinic lithiasis, alkaline mineral waters are preferred [43].

The main moroccan spa resorts recognized in urological treatment are Sidi Harazem, Ain Hamra, Fezouane, and Ain Salama.

### 5.7. Dermatology

The waters used for dermatology treatment contain a chemical element carrying a significant oxidation-reducing power or are rich in hydrogen sulfide (H_2_S) or have calcium and magnesium sulfate or calcium bicarbonate facies.

Traditional indications are rebellious forms of eczema, atopic dermatitis, or psoriasis which concern 80 to 85% of the population received in thermal spas for a dermatological orientation.

Healing disorders are an interesting indication of crenotherapy, although it only affects 2-3% of patients admitted. These are mainly sequelae of burns and some cases of hypertrophic postsurgical scars. Indeed, the realization of a daily mechanical action with aid of soothing and anti-inflammatory thermal water plays a major role in the healing process. All these treatments contribute to reducing inflammation and hypertrophy [44].

The main spa resorts recommended in Morocco for dermatological diseases treatments are Moulay Yaâcoub, Zalagh, Tratt, Tiouka, Ain Boudra, Moulay Driss, Ain Allah, Moulay Ali Cherif, and Abaynou.

### 5.8. Psychosomatic Disorders

Sedative, analgesic, balancing, and relaxing properties of sodium bicarbonate and sulfate waters rich in trace elements contribute to treat some psychosomatic disorders. Different forms of treatments are used for this purpose, such as baths (baths, whirlpools, and underwater shower baths), showers (shower coats, etc.) or special thermal massage “PSY” that relaxes muscles and calms nervous hyperexcitability thanks to the techniques of effleurage, vibration, percussion, and kneading.

The generalized anxiety disorder is improved by the thermal cure. Indeed, treatment with thermal waters proves superior to drug treatment at the eighth week; and improvement is maintained at the sixth month after the cure [45].

The main spas, with this indication in Morocco, are Ain Salama, Fezouane, and Ain Hamra.

### 5.9. Gynecology

In this case, indications of the spa treatment are pelvic pain, hormonal disorders, and sterility. The waters used are generally rich in bromine, iodine, and potassium, which possess decongestant and analgesic properties [30].

The only health resort in Morocco, known for this indication, is Moulay Yaâcoub.

Taken all together, these findings demonstrate that the Moroccan thermal waters could be used as a complementary medicine to relieve pain and in some cases to treat several pathological disorders, especially skin and respiratory diseases. However, there are very few studies based on the principles of evidence-based medicine, demonstrating the therapeutic effects of these Moroccan thermal waters as an alternative to pharmacological therapies. Therefore, more scientific research is necessary to determine these waters' virtues by proving their health beneficial effects.

## 6. Carrying Out Treatment

Various treatment techniques are offered to the visitors of the general public in the Moroccan spas. Some techniques are usual such as baths, swimming pools, showers, vaporarium, footbaths/Maniluves, and jet showers, while others are proposed under medical prescription: lumbar or chest showers and others are offered under medical consultation, especially those concerning ORL (spraying, gargle, nazal irrigation, nebulization, humage, and aerosols).

A brief description of the most used cure techniques in the Moroccan spas will be developed for the treatment of respiratory tract diseases in the most famous concerned one: Moulay Yâacoub. Indeed, the cure techniques used for ORL are “specialized” and based on a contact with the body affected part. Hence, we can use the following:

- Gargle: based mainly on a gentle mechanical cleaning of the oropharyngeal area
- Spray: by directing water on the patient's pharynx and tonsils using a sieve to create a thread-like jet of water, using a palette to divert a water jet, using a mask to enable the inhalation of fine droplets of thermal water, nebulization of large drops of thermal water, or even by nasal irrigation with isotonic water (added with salt)
- Aerosols: by inhaling thermal water fine particles (2 to 5 *μ*m) to reach deeply the respiratory tract, this technique can be coupled with a sonic vibrator to enhance efficiency at the facial sinus level or with pressurization to allow aerosols to enter the Eustachian tube
- Humage: by spraying thermal water in a pressurized porcelain bowl to bathe the patient's nazal cavities and its whole respiratory system [46]

## 7. Conclusion

The richness of Morocco in thermal springs, especially in the Rif, pre-Rif, and SRW regions, combined with its climate mildness and landscape beauty, makes it a favorite place for care and health, to meet the current needs for more natural life and environment. Indeed, several mineral waters from these thermal springs are marketed or used as therapeutic baths to cure some diseases as a main application of the hydrotherapy science. This constitutes the best choice of natural therapy because it uses different water components as only adjuvants and may represent a complement or alternative to conventional drug treatments.

There are more than twenty top-quality therapeutic thermal springs in the country. Thermal waters are used to treat many diseases and pathological problems (respiratory tract troubles, phlebology, gynecology, urinary disorders, digestive disorders, cardioarterial diseases, psychosomatic affections, dermatology, etc.), depending on the source of water. Indeed, the chemical composition and the physical characteristics of a natural mineral water give it scientific therapeutic properties, which are directly related to its underground journey, its depth, the time of transit, and subsoil rocks variety.

These mineral waters are characterized by their biological purity, their chemical stability, and their therapeutic properties revealed by generations of clinicians. Moreover, these waters are used in hydrotherapy, which represents a good alternative in the treatment of many chronic diseases often disabling. Indeed, excellent tolerance, moderate cost, and good compliance related to patient attachment to this therapeutic practice are provided. Therefore, it fits perfectly into the current search for a more natural life and environment, allowing the individual to preserve his health capital more efficiently.

## Figures and Tables

**Table 1:** Physicochemical parameters of some thermal waters in the south Rif (Morocco).

	Outita	Boudra	Tiouka	My Driss	Tratt	Zalagh	Moulay Yaâcoub	Ain Allah	Sidi Harazem	Ain Salama	Fezouane	Sidi Ali
Nature	Source	Source	Source	Source	Source	Source	Source	Forage	Source	Source	Source	Source
*T* (°C)	40	24	24	32	25	37	53	45	33	39	37	52
pH	6.9	7.9	7.03	7.04	7.18	7.1	6.7	7.4	7.4	7.4	7.1	7.1
Cond	11,530	880	45,300	4400	1720	8350	48,250	660	1370	2000	550	
Cl^−^	2197.5	102.95	10,150	447.3	276.9	2094.5	17767.7	69.9	266.9	532.5	33.08	284
SO_3_^2^^−^	917.7	138.8	100.8	720	139.2	192	38.4	67.2	16.8	50.4	26.6	11.22
HCO_3_^−^	268.4	326.35	389.79	414.8	365	292.8	305	366	335.5	34.7		854
Ca^2+^	378.6	111.68	403.5	344.82	51.5	121.29	1434	49.77	80.6	70.54	33.75	108.21
Mg^2+^	111.9	29.61	283.4	93.35	31.8	47.32	419.8	30.5	24.3	32.5		57.39
Na^+^	1277.2	50.79	5161.1	244.67	237.06	1359.3	11315.5	54.6	176.8	310.9		313.5
K^+^	30.9	5.7	41.12	5.56	4.3	25.36	404.5	0.75	2.64			22
Sr^2+^	6.05	0.57	35.56	7.03	2.63	2.5	64.98	0.14	2.2			
SiO_2_	11.4	12.1	9.5	6.6	7.8	8.8	14	6.2	4.5	23.5		
NO^3−^	0.2		26.9	0.2		0.4	15.6		0.3	5.8	3.23	4.96
Li^+^	0.56	0.55	7.54	0.062	0.17	1.16	26.34	0.009	0.03			
Br^−^	3		3.3	1		3	31	0.2	0.5			

My: Moulay; *T* °C: temperature in degrees Celsius; Cond: electrical conductivity: *μ*S/cm indicates that concentrations are in mg/l.

**Table 2:** Main therapeutic orientations of different types of Moroccan mineral waters.

Waters types	Orientations	Thermal sources in Morocco
Sulfurized	Rheumatology ORL Gynecology Dermatology	Moulay Yaâcoub, Zalagh, Tratt, Tiouka, Outita Moulay Yaâcoub, Zalagh Moulay Yaâcoub Moulay Yaâcoub

Sulfated	Rheumatology Dermatology	Moulay Driss, Moulay Ali Cherif, Moulay Hachem. Moulay Driss, Moulay Ali Cherif

Bicarbonate	Respiratory Rheumatology Dermatology Urinary Phlebology	Ain Boudra, Ain Hamra Abaynou, Fezouane, Ain Salama, Ain Hamra Abaynou, Fezouane, Ain Salama, Ain Boudra, Ain Allah SidiHarazem, Fezouane, Ain Salama Abaynou, Ain Hamra

Oligomers	Phlebology Rheumatology Treatment of anemia	Ain Hamra Ain Hamra, Ain Allah Ain Hamra

## Data Availability

Data are available from the corresponding author upon request.
